# Martensitic phase transformation in TiNi

**DOI:** 10.1038/s41598-019-49605-z

**Published:** 2019-09-18

**Authors:** R. Sewak, C. C. Dey

**Affiliations:** 1 Saha Institute of Nuclear Physics, 1/AF Bidhannagar, Kolkata, 700064 India; 2 Homi Bhabha National Institute, Anushaktinagar, Mumbai, 400094 India

**Keywords:** Materials science, Materials for devices

## Abstract

From temperature dependent perturbed angular correlation (PAC) measurements (77–873 K) in equiatomic TiNi intermetallic alloy, martensitic phase transformations have been observed. Three frequency components corresponding to three different phases of TiNi have been found in the temperature range 298–873 K. The results of quadrupole frequency and asymmetry parameters at room temperature are found to be: *ω*_*Q*_ = 14(1) Mrad/s, *η* = 0 (33%), *ω*_*Q*_ = 40.0(5) Mrad/s, *η* = 0.66(3) (52%) and *ω*_*Q*_ = 56.7(3)Mrad/s, *η* = 0.39(2) (15%). The frequency component with *η* = 0 and which enhances to ~52% at 373 K can be attributed to the cubic austenite phase. The predominant component (~52%) found at room temperature has been attributed to monoclinic martensitic phase of TiNi and the third component with values of *ω*_*Q*_ and *η* similar to those for the martensitic phase is attributed to the intermediate orthorhombic phase. At 77 K, no intermediate and austenite phases have been found but only the martensite phase is observed at this temperature. From XRD measurements at room temperature also, three phases of TiNi have been observed.

## Introduction

The TiNi shape memory alloys (SMA) have been widely studied for the last few decades due to its immense technological applications in actuators, robotics, aerospace, condensed matter and in biomedical industries^1–5^. The extraordinary shape memory power of SMA to preserve its original shape under thermal or mechanical stress and also the superelastic properties are exploited in these technological applications. In TiNi SMA, a thermoelastic diffusionless martensitic transformation (MT) occurs at slightly above room temperature which was first observed by Buehler *et al*.^6^. Upon cooling, the SMAs undergo a first order structural transition from the high temperature austenite phase to the low temperature martensite phase. The martensitic transformation is a displacive phase transition and it occurs by coordinated shifts of atoms but, there is no long range diffusion during the phase change. This structural transition can proceed through an intermediate phase. In nearly equiatomic TiNi shape memory alloy, three different phases have been reported as a function of temperature. The high temperature phase is a high symmetry cubic B2 (austenite, space group Pm-3m)^7^. The low temperature phase is a monoclinic B19′ (martensite, space group P2_1_/m)^8^ with a lower symmetry and the intermediate premartensitic R-phase (R) is a hexagonal (space group ${\rm{P}}\bar{3}$)^9^. The structural parameters for different phases reported by Urbina *et al*.^10^ are a = 7.3451 Å, c = 5.2718 Å for the hexagonal R-phase, a = 3.015 Å for the cubic B2 phase and a = 2.898 Å, b = 4.108 Å, c = 4.646 Å, *β* = 97.78° for the monoclinic B19′ phase. The intermediate R-phase transformation is induced by thermal and thermomechanical treatments or by adding a third element in near-equiatomic TiNi alloys^10–13^. The effect of point defects in TiNi based alloy has been intensively studied by doping a third element (Fe, Cu etc.) on Ni site or with excess of Ni to alter the MT temperature and to modify its functional properties^13–25^. The details of TiNi shape memory alloy was reviewed by Otsuka *et al*.^26^ The MT temperature was found to decrease with increase of point defects. It was found that in doped alloy, MT can takes place through the B2-B19-B19′. In this case, the B19 is an orthorhombic phase (space group Pmmb)^25^. The crystal structure properties and formation energies of B2, B19 and B19′ phases were calculated theoretically for TiNi by Huang *et al*.^27^ and for TiNi, TiPd and TiPt by Ye *et al*.^28^.

The equiatomic TiNi was studied earlier by perturbed angular correlation (PAC) spectroscopy^29^ using ^111^In probe. The MT was found near 340 K where, below this temperature, a single quadrupole frequency due to monoclinic structure was found. At T = 400 K, the spectrum was found to be unperturbed due to the structural change to cubic. In the present work, TiNi has been studied by PAC spectroscopy using ^181^Hf probe in the temperature range 77–873 K in order to investigate the structural phase transitions with temperature. The X-ray diffraction studies in TiNi have also been carried out to confirm the phase components.

The perturbed angular correlation is an important nuclear technique^30–32^ for the studies of structural and magnetic phase transition of a material. In this technique, the angular correlation of a *γ*-*γ* cascade of the probe nucleus is perturbed due to the interactions of electromagnetic moments of intermediate level and the electric field gradient or magnetic field generated at the probe site due to surrounding charge distribution. If the probe surrounding charge distribution produces a non cubic symmetry, an electric feld gradient (EFG) is generated at the probe nuclear site.

## Experimental Details

The equiatomic TiNi alloy was prepared by arc melting in argon atmosphere with the constituent elements taken in stoichiometric ratios. The metals in wire forms had purities of 99.99% and 99.98% for Ti and Ni, respectively. The Ti metal was procured from Aldrich and Ni was procured from Alfa Aesar. Repeated melting was carried out to get a homogeneous mixture of the two metals and a shiny globule was formed. No significant weight loss of sample was found and the total sample was ~78.5 mg. With this sample, a pre-activated tiny piece of ^181^Hf metal (~1 mg) was added and re-melted in the arc furnace. This was used for PAC measurements. The Hf concentration in the sample was estimated to be ~0.3 at.%. This small Hf concentration should not influence the sample properties anyway. For activation of ^181^Hf, a natural Hf metal with ~30% ^180^Hf was irradiated by thermal neutrons in the Dhruba reactor using a flux of ~10^13^/cm^2^/s. In a similar way, another TiNi sample was prepared (without active Hf probe). This sample in coarse grain form was used for XRD measurement which was carried out using Rigaku X-ray diffractometer TTRAX-III and Cu K_*α*_ radiation (*λ* = 1.54056 Å).

The daughter ^181^Ta produced in the *β*^−^ decay of ^181^Hf (T_1/2_ = 42.4 days) emits two successive *γ*-rays, 133 keV and 482 keV passing through an intermediate level 482 keV having half-life 10.8 ns and a spin angular momentum *I* = 5/2^+^*ℏ*^33^. The coincidence count rate between these *γ*-rays can be written as

1 $$W(\theta ,t)={\exp }(-t/\tau )[1+{A}_{22}{G}_{22}(t){P}_{2}({\cos }\,\theta )+{A}_{44}{G}_{44}(t){P}_{4}({\cos }\,\theta )]$$

where, *θ* is the angle between the detectors, *τ* is mean lifetime of the intermediate level, P_2_ and P_4_ are Legendre polynomials and A_22_, A_44_ are angular correlation coefficients of the *γ*-*γ* cascade. The perturbation functions G_22_(t), G_44_(t) arise due to hyperfine splitting of intermediate level of the probe nucleus. For the 133–482 keV cascade, A_22_ ≫ A_44_ (A_22_ = −0.288, A_44_ = −0.076)^30^ and, therefore, A_44_G_44_(t) can be neglected. For a polycrystalline material, G_22_(t) is given by^30^

2 $${G}_{22}(t)=[{S}_{20}(\eta )+\mathop{\sum }\limits_{i=1}^{3}{S}_{2i}(\eta )cos({\omega }_{i}t)exp(-\delta {\omega }_{i}t)].$$

Here, *ω*_*i*_ are frequencies related to the hyperfine splitting of the intermediate nuclear level such that *ω*_1_ + *ω*_2_ = *ω*_3_. The EFG tensor *V*_*ij*_ due to electrostatic potential (V) at the nucleus are given by

3 $${V}_{{x}_{i}{x}_{j}}={{\rm{\partial }}}^{2}V/{\rm{\partial }}{x}_{i}{\rm{\partial }}{x}_{j},\,{x}_{i},{x}_{j}=x,y,z,$$

In the principal axis system, only the diagonal components are non-zero and ∇^2^*V* = 0. Thus, EFG is conventionally expressed by largest diagonal component *V*_*zz*_ and the asymmetry parameter defined as

4 $$\eta =\frac{({V}_{xx}-{V}_{yy})}{{V}_{zz}},\,0\le \eta \le 1$$

and the quadrupole frequency *ω*_*Q*_ is related to *V*_*zz*_ by

5 $${\omega }_{Q}=\frac{eQ{V}_{zz}}{4I(2I-1)\hslash }$$

where, Q is nuclear electric quadrupole moment of the intermediate level. For *I* = 5/2^+^ and *η* = 0 (axial symmetry), there are three observable frequencies *ω*_1_, *ω*_2_ and *ω*_3_ related as

6 $${\omega }_{Q}={\omega }_{1}/6={\omega }_{2}/12={\omega }_{3}/18$$

The defination of S_*kn*_ coefficients can be found in ref.^34^. Due to chemical inhomogeniety in the sample, all probe nuclei do not experience exactly the same EFG but slightly different EFG’s are experienced which produces a distribution of frequency around the principle value and this gives rise to a broadening of frequency. Here, a Lorentzian frequency distribution is considered in the perturbation function where, *δ* is the frequency distribution width. The experimental setup of PAC is equipped with two LaBr_3_(Ce) detectors (38 × 25.4 mm^2^) and two BaF_2_ detectors (50.8 × 50.8 mm^2^). Four standard slow-fast coincidence combinations were formed to acquire four coincidence spectra at 180° and 90° The LaBr_3_(Ce) was used for the selection of 133 keV *γ*-rays and the 482 keV *γ*-rays were selected in BaF_2_ detectors. An instrumental prompt time resolution (FWHM) of ~653 ps has been found for the LaBr_3_(Ce)-BaF_2_ set up using a ^22^Na source and selecting the 133 and 482 keV *γ*-ray energies. From the measured coincidence counts at 180° and 90°, a ratio R(t) is formed. This is given by

7 $$R(t)=\frac{2}{3}[\{\sqrt{\frac{{W}^{13}({180}^{\circ },t){W}^{24}({180}^{\circ },t)}{{W}^{14}({90}^{\circ },t){W}^{23}({90}^{\circ },t)}}\}-1]$$

Here, *W*^13^(180°, *t*) is the random subtracted coincidence count for the detector combination of 1 and 3 set at an angle 180° at time channel t. Neglecting A_44_ (A_22_ ≫ A_44_), the *A*_22_*G*_22_(*t*) can be found from R(t) as^34^

8 $${A}_{22}{G}_{22}(t)=\frac{R(t)}{\{1+R(t)/2\}}$$

## Results and Discussion

**Figure 1:**
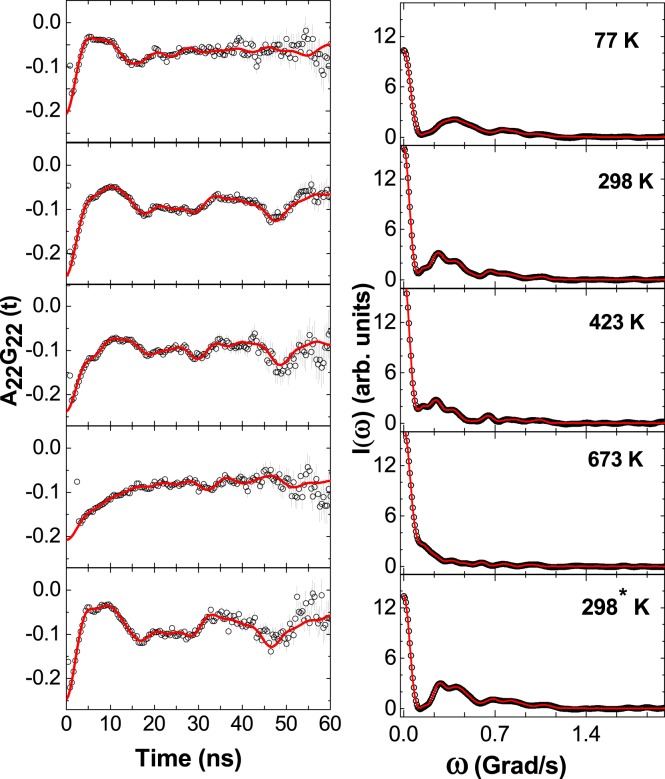
TDPAC spectra of TiNi at different temperature. Left panel shows A_22_G_22_(t) vs time(t) and right panel shows corresponding Fourier Transformation. The spectrum designated by 298^*^ K was taken after measurement at 723 K.

The results of PAC measurements in TiNi are shown in Table 1 and the corresponding spectra at selected temperatures are shown in Fig. 1. The three electric quadrupole frequencies are observed at room temperature and above (up to 873 K). After first thermal cycle, the dominating component (52%) gives *ω*_*Q*_ = 40.0(5) Mrad/s, *η* = 0.66(3) and *δ* = 12(1)% at room temperature (component 2). This component can be assigned as monoclinic martensite phase (M) of TiNi. The corresponding electric field gradient for the monoclinic phase is found to be V_*zz*_ = 4.5 × 10^21^ V/m^2^.

**Table 1:** Results of PAC measurements in the TiNi sample after first thermal cycle.

Temperature (K)	Component	*ω*_*Q*_(Mrad/s)	*η*	*δ*(%)	*f*(%)	Assignment
298^†^	1	14.3 (7)	0	0	27 (2)	TiNi (A)
	2	32 (2)	0.55 (5)	25 (7)	66 (3)	TiNi (M)
	3	57.1 (7)	0.37 (6)	0	7 (2)	TiNi (I)
77	2	39.8 (7)	0.72 (3)	18 (2)	83 (3)	TiNi (M)
	4	4.0 (1)	0	0	9 (2)	
	5	45.6 (8)	0	0	8 (2)	
298	1	14 (1)	0	0	33 (2)	TiNi (A)
	2	40.0 (5)	0.66 (3)	12 (1)	52 (3)	TiNi (M)
	3	56.7 (3)	0.39 (2)	0	15 (2)	TiNi (I)
423	1	14.2 (3)	0	0	48 (3)	TiNi (A)
	2	42 (1)	0.63 (11)	8 (3)	31 (2)	TiNi (M)
	3	54.9 (6)	0.39 (4)	0	20 (2)	TiNi (I)
523	1	13.7 (2)	0	0	59 (4)	TiNi (A)
	2	41.3 (7)	0.66 (5)	0	14 (3)	TiNi (M)
	3	53.8 (3)	0.46 (3)	0	27 (3)	TiNi (I)
623	1	12.8 (2)	0	0	57 (3)	TiNi (A)
	2	42.2 (7)	0.48 (6)	0	18 (2)	TiNi (M)
	3	52.1 (3)	0.44 (2)	0	25 (2)	TiNi (I)
723	1	12.0 (2)	0	0	57 (3)	TiNi (A)
	2	43.3 (5)	0.40 (3)	0	20 (2)	TiNi (M)
	3	51.1 (4)	0.46 (2)	0	23 (2)	TiNi (I)
298^*^	1	14.9 (1)	0	0	16 (2)	TiNi (A)
	2	42.0 (4)	0.65 (2)	11 (1)	66 (3)	TiNi (M)
	3	58.0 (3)	0.42 (3)	0	18 (2)	TiNi (I)

^†^In as synthesized sample while ^*^in same sample after second thermal cycle.

**Figure 2:**
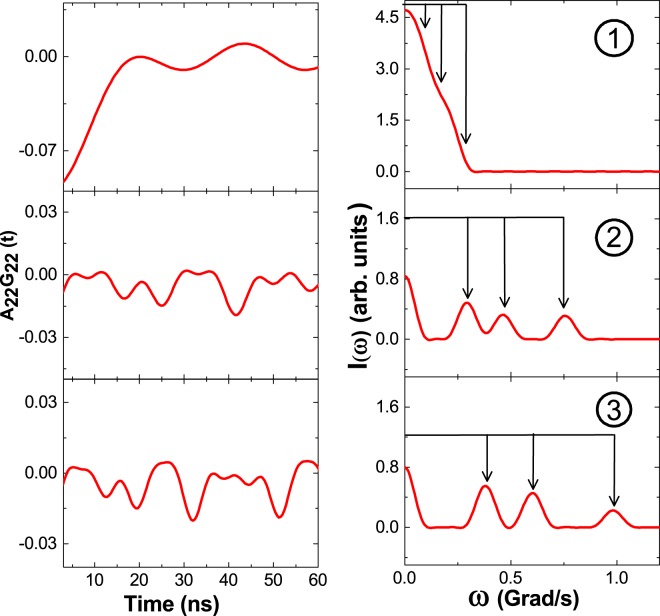
Decomposed TDPAC spectra for components 1, 2 and 3 in TiNi at 673 K.

**Figure 3:**
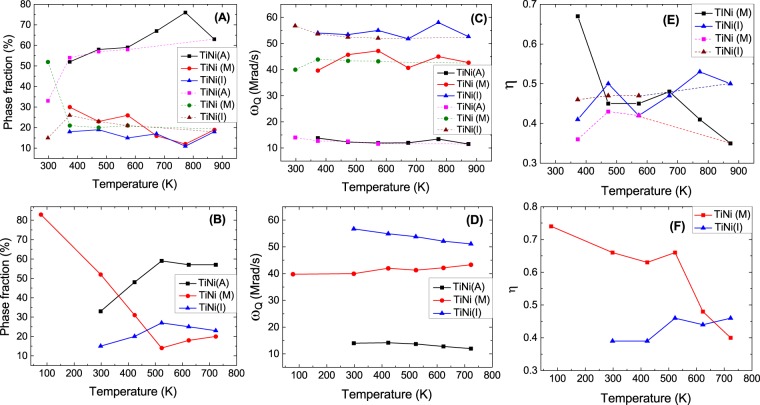
Variations of phase component fraction (%) are shown in (**A**,**B**), quadrupole frequency (*ω*_*Q*_) are shown in (**C**,**D**), asymmetry parameter (*η*) are shown in (**E**,**F**). In top spectra, solid lines represent forward cycle and the doted lines represent reverse cycle. The spectra at bottom correspond to the second thermal cycle.

The first component with values of *ω*_*Q*_ = 14.8(1) Mrad/s, *η* = 0 has site fraction ~19% at room temperature which increases to 52% with values of *ω*_*Q*_ = 13.8(3) Mrad/s, *η* = 0 at 373 K. Results of *ω*_*Q*_ and *η* for this component indicate a cubic structure and an abrupt increase of this component at 373 K at the expense of the martensite phase further suggests this as a cubic austenite phase (A). In second thermal cycle, this component is found to be ~48% at 423 K with almost same values of *ω*_*Q*_ and *η* as found at 298 K. The probe Ta atoms can experience non-zero value of EFG in the cubic TiNi lattice if these are positioned in a slightly distorted lattice environment. A third component with values *ω*_*Q*_ = 56.7(3) Mrad/s, *η* = 0.39(2), *δ* = 0 are found at room temperature (Table 1) and it is found to be present in the temperature range 77–873 K. This value of *ω*_*Q*_ corresponds to V_*zz*_ = 6.3 × 10^21^ V/m^2^. Three decomposed components at 673 K are shown in Fig. 2. Components 1 and 2 show strong texture effects which means the sample is not perfect polycrystalline (crystallites are not randomly oriented). Therefore, we have considered free S-coefficients for analysis of data. Variations of *ω*_*Q*_, *η* and percentage site fractions (f) with temperature for different components are shown in Fig. 3. Our results show that for component 3, the site fraction does not change appreciably with temperature. The austenite phase of TiNi (component 1) increases with temperature at the expense of component 2. The component 3 can, therefore, be attributed to the intermediate phase (I). At 77 K, we have found only the martensitic phase of TiNi (Table 1). The two minor components found at this temperature with low site fractions are, probably, due to trapping of defects. Here, no cubic austenite phase is observed. Therefore, from present PAC measurement, the austenite start temperature is found at ~298 K.

The crystal structure of the intermediate R-phase was reported to be hexagonal (space group ${\rm{P}}\bar{3}$) by Urbina *et al*.^10^. But, a definite value of *η* for the intermediate phase found from present PAC measurements indicates that structure of intermediate phase is not hexagonal. Rather, closer agreements of results for the monoclinic TiNi and the intermediate phase suggest these two have structural similarities. A hexagonal structure is expected to produce asymmetry parameter *η* = 0. Here, the intermediate phase can be assigned to the orthorhombic B19 structure (space group Pmmb). The intermediate R-phase was found in TiNi^10^ and in Ti-Ni-Fe system^13^ while the B19 intermediate phase was found in ternary systems with Ti-Ni-Cu and Ti-Ni-Pd^25^. Our PAC measurements in TiNi, on the other hand, suggest that the intermediate phase has orthorhombic B19 structure. The crystal structure parameters reported for the R-phase^10^ were found to be quite different from the B19 phase^25^. So, if the intermediate phase was R, PAC results for components 2 and 3 would be quite different. But, similar values of *ω*_*Q*_ and *η* found for these components indicate that intermediate phase is not R. Otsuka *et al*.^35^ reported that monoclinic B19′ structure is derived from B2 in two steps. Namely, the structure B2 transforms into a B19 first, and then a monoclinic martensite is derived by shearing the B19 in [100]_*B*19_ direction on (001)_*B*19_ plane. However, the B19 structure in TiNi was not reported earlier although this structure was found to be stabilized in TiPd and TiPt. The results from present temperature dependent PAC measurements suggest that TiNi has three crystal structures in the temperature range 298–373 K.

The results from present PAC measurements using ^181^Hf and the results from previous measurements using ^111^In probe^29^ give the similar MT values. However, there are differences between these results- (i) a single frequency component corresponding to monoclinic TiNi phase was observed from the previous PAC measurement^29^ at room temperature while three frequency components have been found from present measurements and ii) only a single quadrupole frequency with a broad frequency distribution was found from previous measurement^29^ at 400 K which was attributed to the cubic austenite phase. At 400 K and above, no monoclinic martesite phase and also no intermediate phase was observed. From present measurements, all three frequency components have been observed up to 873 K with changes in relative fractions but, no pure austenite phase has been found up to 873 K.

**Figure 4:**
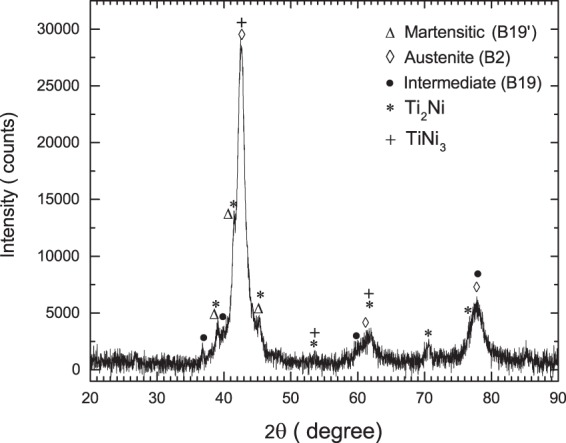
Powder XRD pattern of TiNi at 298 K in as synthesized sample.

**Figure 5:**
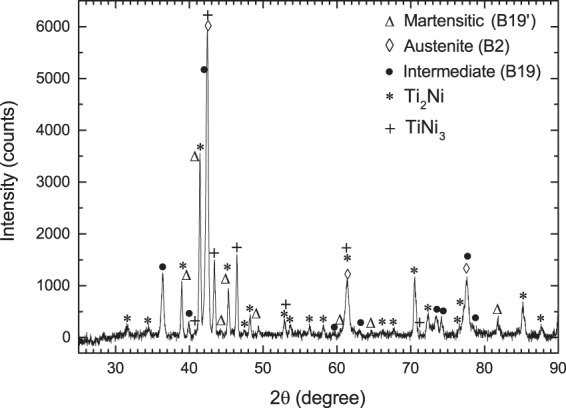
Powder XRD pattern of TiNi at 298 K after annealing the sample at 873 K for ~45 hrs.

The XRD powder diffraction patterns for TiNi in both as prepared and annealed sample are shown in Figs 4 and 5. The sample annealed at 600 °C for ~45 hrs gives a much better XRD pattern compared to that found in pre-annealed sample. The different peaks found in annealed sample are identified as indicated. The peaks corresponding to three phases of TiNi viz. monoclinic B19′ phase^8^, cubic B2 phase^7^ and intermediate phase are found. The intermediate phase is found to be B19 instead of R-phase, by comparing the present XRD pattern of annealed sample (Fig. 5) with the XRD patterns generated for R-phase^9^ and orthorhombic B19 phase^25^. From our PAC measurement also, the R-phase for the intermediate TiNi phase is not supported. The reason for getting B19 phase instead of R-phase from present measurements is not understood where it was found from previous measurements that presence of a third element (Cu/Pd) only induced the B19 phase^25^. Apart from these, the peaks corresponding to Ti_2_Ni and TiNi_3_ are clearly found in annealed sample. Here, presence of Ti_2_Ni (space group Fd-3m^36^) and TiNi_3_ (space group P6_3_/mmc^37^) can be explained by considering the eutectoid decomposition of TiNi to Ti_2_Ni and TiNi_3_ as reported earlier^38^. Indication of these two phases are found also in preannealed sample and these are probably produced during sample preparation in arc furnace. From PAC measurements, however, no signal due to Ti_2_Ni or TiNi_3_ are found at any temperature. The component 3 with a finite value of *η* can not be assigned to Ti_2_Ni (cubic structure) or TiNi_3_ (hexagonal structure). The wt% of these two phases were probably small in our PAC sample and could not be detected.

## Conclusion

From present investigations, three crystalline phases of TiNi viz. cubic austenite B2, monoclinic martensite B19′ and intermediate orthorhombic B19 have been found in the temperature range 298–873 K. The B19 orthorhombic structure has been found as an intermediate phase in the transformation from cubic B2 to monoclinic B19′ phase. A martensitic phase transformation at ~298 K has been found in this material. The austenite phase is found to increase with temperature at the expense of the martensitic phase. At 77 K, only monoclinic phase of TiNi is found. In present sample, no R–phase is observed. From present studies, observation of a stable B19 phase co-existing with B19′ phase in TiNi, not reported earlier, is interesting and needs further investigations both theoretically and experimentally. Weak temperature dependent variations of *ω*_*Q*_ for all three phases indicate that individual crystal structures do not change much with temperature. The results for *ω*_*Q*_ and phase fractions depend slightly on the thermal history of the sample. From previous PAC measurements using ^111^In probe, however, only the monoclinic phase below the transition temperature (~340 K) with a single quadrupole frequency and a cubic austenite phase above this temperature was found. Observed phases of Ti_2_Ni and TiNi_3_ from present XRD measurements, probably, give a clear experimental evidence of eutectoid decomposition of TiNi to Ti_2_Ni and TiNi_3_.
